# Connectomes: a re-emerging hub dataset for neuroscience

**DOI:** 10.3389/fnins.2026.1911198

**Published:** 2026-09-29

**Authors:** Sebastian Kyllmann, Ian W. Keesey

**Affiliations:** School of Biological Sciences, University of Nebraska-Lincoln (UNL), Lincoln, NE, United States

**Keywords:** connectomics, *Drosophila*, network neuroscience, neural circuits, review, structure-function

## Abstract

Connectomics aims to generate complete neural wiring diagrams and leverage them to explain brain function at a systems level. Traditional neuroscience has relied on sparse recordings and partial circuit reconstructions, limiting mechanistic insight into how distributed neural activity produces behavior. By contrast, connectomes provide comprehensive maps of all neurons and all synapses, analogous in some respects to the transformative impact of whole-genome sequencing in biology. Early efforts, including the *Caenorhabditis elegans* connectome, required extensive manual reconstruction, but recent advances in electron microscopy, light-based imaging, and artificial intelligence now enable large-scale datasets such as the full nervous system connectomes of *Drosophila melanogaster*. These datasets are increasingly integrated into experimental and computational research to define neuronal cell types, reconstruct fine-scale circuits, and analyze network organization. Connectomics has enabled the discovery of novel neuron types, clarified sensory and motor pathways, and facilitated predictive models of neural computation. Beyond anatomical reconstruction, connectome graphs increasingly serve as substrates for network analysis, simulation, and perturbation, providing a framework for generating and testing mechanistic hypotheses about brain function. Network studies reveal conserved principles, including small-world topology and rich-club organization, while connectome-constrained simulations and perturbation approaches offer new opportunities for linking structure to function. Despite these advances, connectomes remain limited by small sample sizes, scale, cost, and their fundamentally static nature, lacking direct information on neural dynamics, physiological state, and plasticity. Integrating structural data with functional, neuromodulatory, glial, and behavioral measurements remains a major challenge. Nonetheless, connectomes have emerged as foundational hub datasets that organize diverse streams of neuroscientific evidence and provide an increasingly important framework for advancing a mechanistic understanding of brain function and dysfunction.

## 1 Introduction

We are living in an exciting time for neuroscience with the accelerated publication of whole brain maps for increasingly complex species. Termed connectomes, these three-dimensional maps track every neuron and every synapse in an animal’s brain. Whereas prior neuroscience was necessarily restricted to small parcels of the brain and discrete neural circuits, future neuroscience may use the roadmap provided by connectomics to tackle questions about function across the whole nervous system (Figure 1). Although analogies between neural circuits and electronic circuits are imperfect and often controversial, they nonetheless highlight an essential point: without a detailed wiring diagram of a complex engineered system such as a microprocessor, meaningful progress in reverse-engineering its function would be impossible (Jonas and Kording, 2017). Neuroscience, by contrast, has long attempted to infer brain function without an equivalent wiring diagram, relying instead on sparse measurements, limited perturbations, and incomplete anatomical data. This absence of a complete circuit description has constrained our ability to explain how neural systems process information.

**FIGURE 1:**
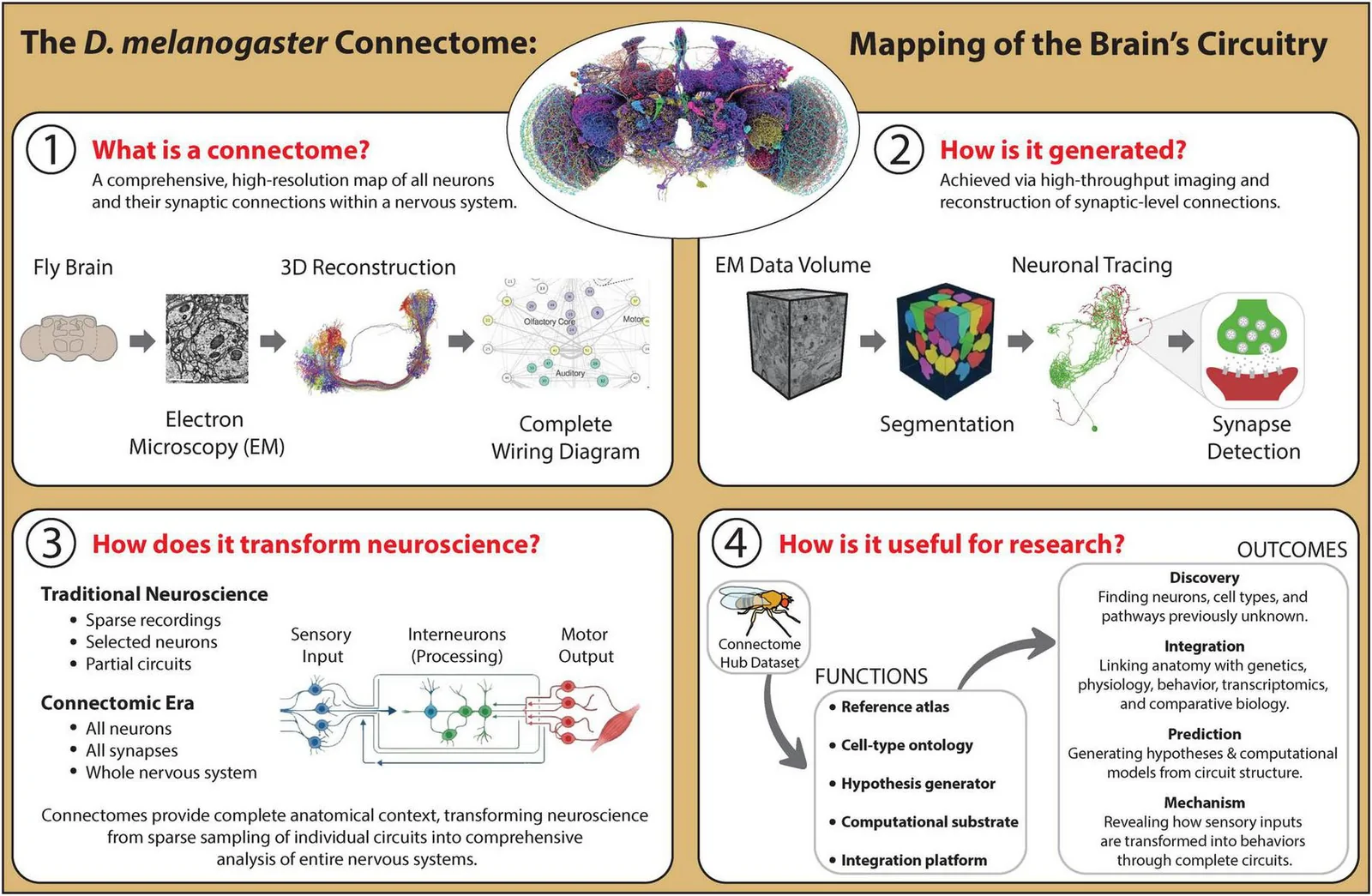
Connectomes as a hub dataset for neuroscience. A connectome is a comprehensive map of neuronal and synaptic connectivity generated through large-scale imaging and reconstruction pipelines. By providing complete anatomical context at the scale of entire nervous systems, connectomes transform neuroscience from the study of selected neurons and partial circuits to systems-level analyses of neural organization and function. Connectomes now serve as central hubs, promoting functional resources while generating important outcomes across the field of neuroscience.

In this context, whole-brain connectomes represent the neuroscience analog of genomic datasets: foundational resources that may unlock new explanatory frameworks, much as inexpensive genome sequencing transformed genetics and molecular biology. Where early genetic research was limited to a handful of genes, next-generation sequencing enabled the rapid, cost-effective decoding of entire genomes, transforming biology into a data-rich, systems-level science. Currently, even simple brains of model species remain a black box, where sensory inputs and behavior outputs undergo complex processing not fully understood by neurobiologists. Thus, connectomes can be viewed as necessary datasets, but not sufficient to fully understand the complete workings of the nervous system.

The publishing of the very first connectome, for the nematode *Caenorhabditis elegans*, happened nearly 40 years ago (White et al., 1986) and represented the culmination of a decade of effort to manually reconstruct the *C. elegans* nervous systems from electron microscopy (EM) sections. These early efforts involved skilled researchers tracing neurons and identifying synapses using felt tip pens on printed EM images. In the intervening decades, only a few full connectomes were published including the larval stages of sea squirt *Ciona intestinalis* (Ryan et al., 2016) and the marine annelid *Platynereis dumerilii* (Verasztó et al., 2025). These full connectomes were complemented by partial connectomes of important nervous system substructures in organisms such as the fruit fly *Drosophila melanogaster* (Berck et al., 2016; Eichler et al., 2017; Li et al., 2020; Scheffer et al., 2020; Hulse et al., 2021), *C. elegans* (Jarrell et al., 2012), the mouse *Mus musculus* (Helmstaedter et al., 2013; Kasthuri et al., 2015; Lee et al., 2016; The MICrONS Consortium et al., 2025), the zebra finch (Kornfeld et al., 2017; Shvedov et al., 2026), the zebrafish (Wanner et al., 2016; Hildebrand et al., 2017; Svara et al., 2022), the octopus (Bidel et al., 2023) and even humans (Shapson-Coe et al., 2024). These combined efforts of resuscitating the grand goal of comprehensive nervous system maps culminated with the publication of the full central nervous system connectomes of larval and adult *D. melanogaster* (Zheng et al., 2018; Winding et al., 2023; Dorkenwald et al., 2024), nearly 40 years after the first *C. elegans* connectome. Like the first connectome, these complete connectomes required a decade of sustained efforts. Innovations in microscopy and artificial intelligence technology allowed the reconstruction of a *D. melanogaster* nervous system with 400 times the number of neurons and 7,000 times the number of synapses as *C. elegans*. These innovations have also reinvigorated the field of connectomes, with new comprehensive connectomes for nematodes (Cook et al., 2019, 2025), the larval zebrafish (Svara et al., 2022), and *Drosophila melanogaster* (Takemura et al., 2023; Azevedo et al., 2024; Berg et al., 2025; Bates et al., 2026). Despite these promising advances across organisms, we mostly restrict this review to *D. melanogaster* due to the complexity of their behavioral repertoire, the nervous system-wide scale of their connectomes, and the increasingly rapid publication of these datasets which have moved beyond solitary examples in recent years. In Figure 2 we summarize the connectomes available at the time of writing this review and common acronyms used for these datasets in both this review and the broader literature. Because connectomics is advancing rapidly, this review also discusses recent preprints and community datasets that have not yet completed peer review but are already shaping experimental and computational neuroscience.

**FIGURE 2:**
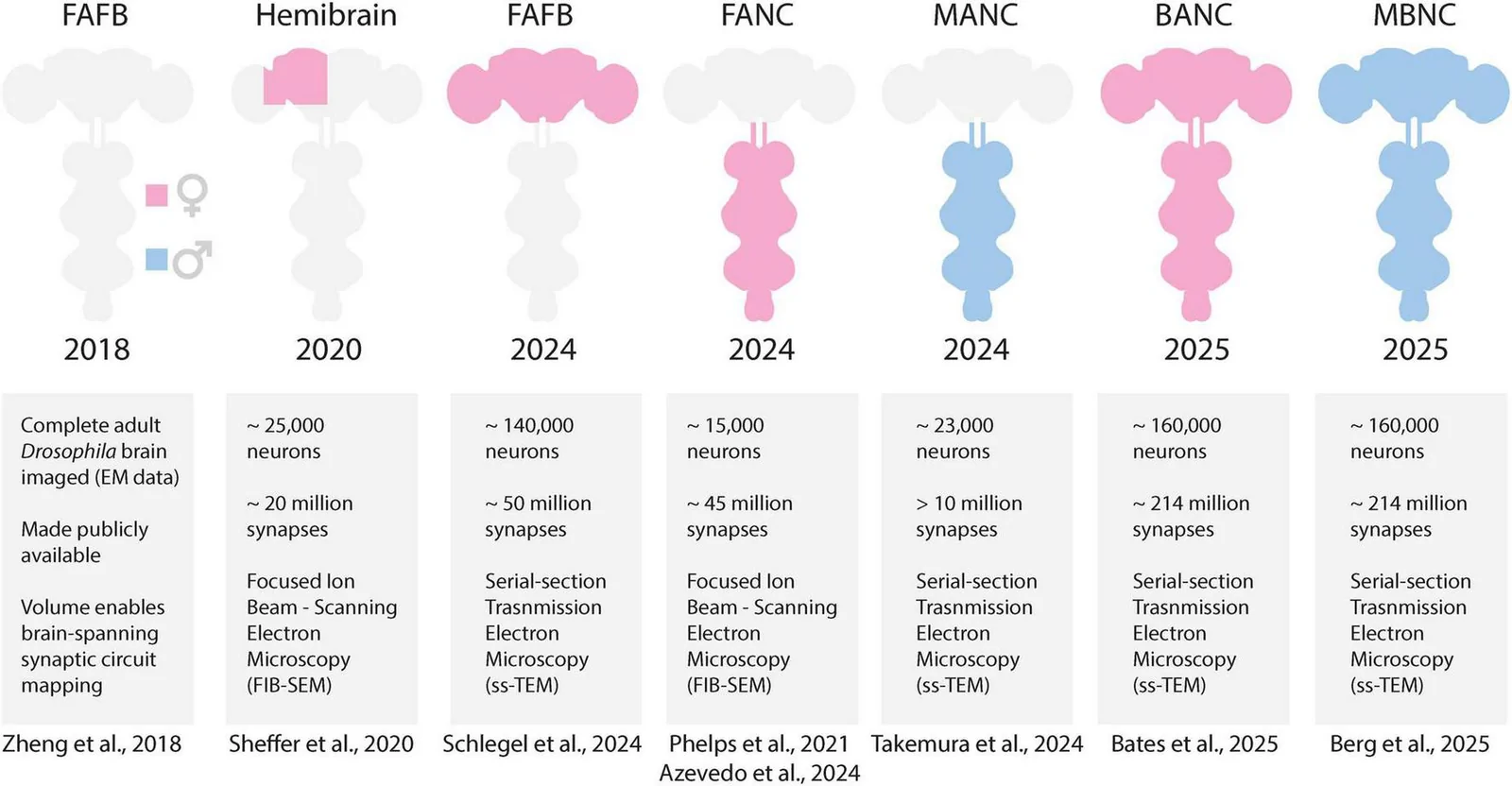
Overview of timelines and *Drosophila melanogaster* connectomes. Females individuals shown in pink, and males shown in blue. First electron microscopy (EM) scans made public in 2018, then the rapid mapping of neurons and synapses are published, first for female, then male. FAFB, full adult fly brain; FANC, full adult nerve cord; MANC, male adult nerve cord; BANC, brain and nerve cord, female; MBNC, male brain and nerve cord.

Continued advances in microscopy and artificial intelligence will likely accelerate the number of published connectomes in the next few years. This trajectory parallels the explosive growth in genomics that followed the advent of high-throughput sequencing technologies. Recent breakthroughs in high-resolution light-based imaging, neuron barcoding, volume electron microscopy, and machine learning-based segmentation and proofreading are beginning to make whole-brain connectome reconstruction feasible at scale. As sequencing technology did for genomics, these tools are lowering the technical and financial barriers to connectome mapping, thus laying the groundwork for integrative neural circuit analysis across species, developmental stages, and disease states. Understanding the full connectivity of multiple individuals, different sexes, or closely related species, and that of increasingly complex nervous systems (e.g., vertebrates) will allow scientists to better answer questions about how brains have evolved throughout natural history and how complex behaviors are generated by the brain. Insights gained from the connectomics of model species will likely yield critical insights concerning neurological and psychiatric diseases. Diseases such as Alzheimer’s, autism, and schizophrenia involve pathological disruptions to brain connectivity, and these conditions have been tentatively described as connectopathies.

Nonetheless, significant scaling challenges remain in mapping mammalian brains due to the power law scaling of animal nervous systems. Although *D. melanogaster*, mice and humans possess similar ranges of protein coded genes (13,000 to 20,000), the mouse and human brain respectively contain between 700 to 850,000 times more neurons and 1,000 to 10,000,000 times more synapses than the *D*. *melanogaster* brain. Furthermore, although necessary, connectomes are ultimately not sufficient for understanding the relationship between brain connectivity and nervous system function. They represent a static picture of a particular brain at a fixed moment in time, thus lacking any information of synaptic rewiring over the course of an organism’s lifetime. Even for the 300-neuron nervous system of *C. elegans* there are only a handful of published connectomes, limiting our understanding of how wiring differs between individuals, sexes, ages, experience, learning, and genetic backgrounds of animals belonging to the same species.

Finally, understanding the dynamic function of the brain will require connectomic datasets to move beyond structural data alone. Synapse counts do not translate directly into functional weights, and wiring diagrams cannot by themselves specify neuronal dynamics, neuromodulatory state, electrical coupling through gap junctions, synaptic plasticity, membrane excitability, metabolic constraints, or the behavioral context in which a circuit operates. Future connectomic frameworks will therefore need to be integrated with complementary measurements of non-synaptic processes, including neuromodulatory signaling, glial physiology, gene expression, physiological state, and *in vivo* neural activity. Emerging technologies may even enable *in vivo* circuit rewiring experiments, allowing structural and functional hypotheses to be tested directly against one another. Beyond neurons, a major frontier for comparative connectomics will be the incorporation of glial cells and other non-neuronal elements into circuit reconstructions. Astrocytes, microglia, oligodendrocytes, and related cell types are now recognized as active participants in circuit development, synapse formation and elimination, plasticity, metabolic support, myelination, and network homeostasis, rather than passive structural support cells (Allen and Lyons, 2018). Their organization and physiological state may vary substantially across individuals as a function of development, experience, aging, and disease, potentially contributing to functional differences that are not evident from neuronal wiring diagrams alone. Recent work has highlighted the broad and dynamic roles of glia in shaping nervous system function. In particular, astrocytes actively regulate synaptic transmission and plasticity through bidirectional signaling with neurons (Fields et al., 2015), suggesting that a complete understanding of circuit operation will require mapping interactions among neuronal, glial, and other non-synaptic components of neural systems. Integrating these features into future connectomes may help explain how individuals with similar neuronal architectures nevertheless exhibit substantial differences in circuit function, behavior, cognition, and susceptibility to neurological disease.

Despite any of the mentioned limitations, connectomes are increasingly functioning as central hub datasets within neuroscience. Here we define a hub dataset as a single foundational resource that is reused across multiple scientific roles or disciplines, including anatomical reference, cell-type classification, circuit reconstruction, computational modeling, hypothesis generation, and cross-modal data integration. As with reference genomes in biology, the value of a connectome lies not only in the dataset itself, but in its ability to organize and connect diverse forms of scientific evidence. Rather than diminishing the value of connectomes, any constraints or limitations have simply shaped how they are currently used, and driven efforts to refine them going forward. This review argues that connectome datasets have already become a central and versatile resource in *Drosophila* neuroscience, generating many powerful new avenues for major research efforts. Importantly, *D. melanogaster* connectomes now provide a critical lens through which larger vertebrate connectomes may be approached and interpreted. Many of the analytical frameworks, computational models, and circuit-level hypotheses first developed in flies may increasingly inform connectomic studies in fish, avian, rodent, and ultimately human nervous systems, allowing *D. melanogaster* to function as both a model system and a proving ground for the future of systems neuroscience. Here, the fly connectomes and their role is best understood along two dimensions. First, functionally, they serve as anatomical references, as cell-type ontologies, as generators of circuit hypotheses, as substrates for computational modeling, and as platforms for cross-dataset integration (Figure 3). Second, evidentially, the weight they carry varies across studies, from core datasets that are consulted mainly as references, to datasets that validate or contextualize experimental findings, or to datasets from which functional predictions are generated and subsequently tested (Figure 3). Together, these two dimensions of connectome use reveal a neurobiology field in transition, namely that connectomes are increasingly used not just to look up what is connected and how, but to predict what a circuit does and, in the strongest cases, to test that prediction through function or modeling of the nervous system at the level of the whole organism.

**FIGURE 3:**
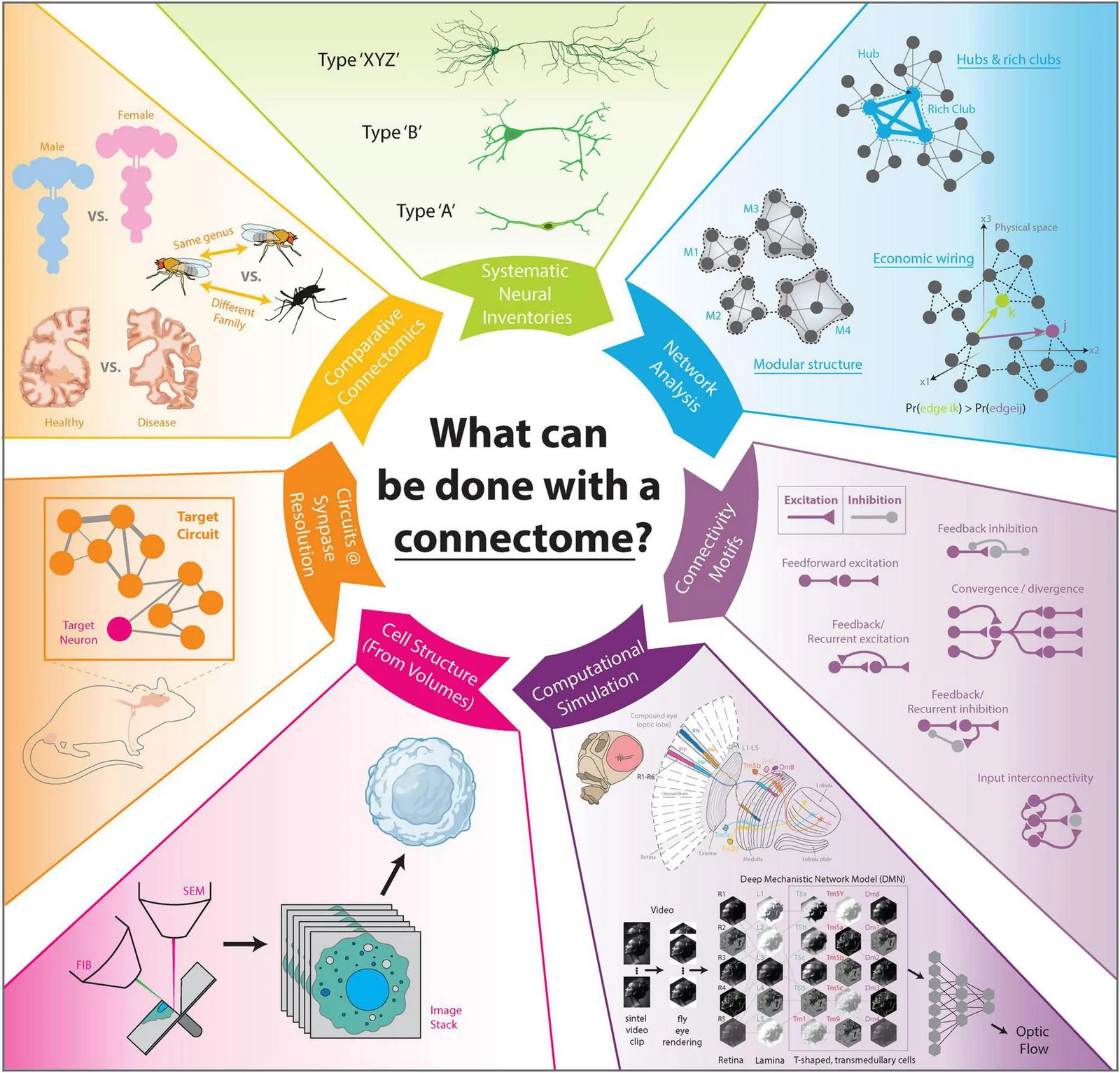
Summarized framework of this review, highlighting connectomes as a central hub for many discoveries. Here we identify several opportunities for connectomes to fundamentally change our understanding of the brain, from functional units to organizational principles, as well as across sex, age, physiological states, and species boundaries. Some images recreated by authors and adapted from Galili et al. (2022), Lappalainen et al. (2024), Shiu et al. (2024).

## 2 Systematic neuronal inventories

The most basic function of a connectome is to act as a reference catalog, providing a systematic inventory of all neurons in the nervous system of a given organism. This function has benefited from intuitive graphical interfaces such as neuPrint (Plaza et al., 2022) and Codex (Dorkenwald et al., 2024) which permit researchers to easily search, summarize, and visualize neurons of interest. Many neurons will have been tested experimentally and characterized for function in a pre-connectomics era, yet many will likely remain unstudied or understudied. Thus, connectomes can provide new targets for experimental characterization or reinvigorate studies of previously described neurons with novel brain-wide context. Overall, a comprehensive anatomical dataset such as a connectome allows researchers the ability to search across the breadth of the nervous system for known targets, or new sites for study.

However, the true power of brain-wide neuronal inventories is the ability to systematically classify neurons into discrete units that may range from one to over a thousand neurons. The biological concept of cell types directly lends itself to this task. These cell types may be classified by functional, morphological, developmental or transcriptomic properties (Allen et al., 2020). These methods are critical for reducing the dimensionality and understanding the function of nervous systems with hundreds of thousands of neurons such as *D. melanogaster*. As vertebrate connectomes become feasible to reconstruct, cell typing may become even more valuable to reduce the dimensionality of these larger nervous systems.

The appropriate amount and composition of cell types will likely remain a topic of discussion going forward in connectomics. Cell typing will thus be distinguished between “lumpers” and “splitters,” analogous to other scientific disciplines. For theoretical neuroscientists, a binary classification of neurons into excitatory and inhibitory may be most appropriate. Yet for experimentalists, smaller groups of neurons will likely be most useful for experimental characterization. Cell types will thus be most useful when they overlap to a large extent with existing genetic resources such as the FlyLight split-GAL4 lines (Meissner et al., 2025; Shuai et al., 2025). In the GAL4/UAS system, a driver line expresses the transcription factor GAL4 under a defined enhancer, and an effector transgene downstream of a UAS sequence is expressed only in those cells; split-GAL4 refines this further by reconstituting functional GAL4 only where two separately driven hemidrivers (an activation and a DNA-binding domain) overlap, yielding near-single-cell-type specificity. Such lines allow the insertion of experimental tools such as optogenetic silencing and activation to test the functional role of these cell types. Tools such as NeuronBridge (Clements et al., 2024) bridge the two data modalities by indexing EM and LM imagery and returning cross-modal morphological matches, so that a neuron identified in an EM connectome of the fly can be linked to candidate FlyLight driver lines targeting it.

The greatest beneficiary of cell typing enabled by connectomes has been the visual system in *Drosophila melanogaster*, given the repeated columnar structure of the optic lobes and the subsequent ability to reduce 10,000 optic neurons into only a few hundred cell types (Nern et al., 2025). The specific advantage that connectomes provide for cell typing is the ability to systematically define connectivity-based cell types (Matsliah et al., 2024; Schwartzman et al., 2026). This method of cell typing complements prior methods but may also reveal novel cell types, with the recently published male, right optic lobe revealing around 350 new visual neuron cell types when compared to previous literature (Nern et al., 2025).

The power of connectome-enabled cell typing is best illustrated by the example of Dm3 neurons. These neuron types of the *D. melanogaster* optic lobe were previously described and subdivided into two subtypes (Strausfeld, 1970; Fischbach and Dittrich, 1989; Nern et al., 2015). Connectivity based cell typing added a third subtype (Matsliah et al., 2024), which was key for predicting the function of Dm3 neurons (Seung, 2024). Where Dm3 neurons were previously visualized as randomly scattered axons across the optic lobe, each of the three Dm3 subtype axons were now shown to striate along a hexagonal axis of the compound eye (Seung, 2024). By analyzing connectivity with three TmY neurons, a circuit for form vision (the neural processing of shapes and edges) was predicted from connectomics alone (Seung, 2024). Thus, in this case connectomes were necessary and particularly powerful for generating a novel hypothesis regarding Dm3 neuron subtype function, which was subsequently verified through *in vivo* functional recordings (Currier and Clandinin, 2025).

Another powerful example of connectome-enabled cell typing relates to the previously unknown LT52 neurons, a novel visual cell type revealed by the connectome (Hartman et al., 2025). Here the connectome did not merely corroborate an existing physiological picture but supplied the object of study itself. LT52 was first delineated in the full-brain connectome annotation, and connectome-based image matching was even used to identify driver-line reagents for genetic targeting. Subsequent *in vivo* recordings of LT52 neurons showed them responding more robustly to internal movement signals, with weaker activation by oblong visual stimuli (Hartman et al., 2025). Critically, nearly every hypothesis these recordings tested was itself connectome-derived, and along an interpretable input/output axis. Tracing LT52’s inputs generated predictions about the origin of its activity: a short, three-synapse pathway from foreleg proprioceptors through ascending neurons and visual centrifugal cells (notably cLLPM02), reconstructed only by cross-referencing the whole-brain, BANC, and male nerve-cord connectomes, alongside dominant visual input from the object-motion cell LC10. Tracing its outputs, meanwhile, generated the paper’s central hypothesis about function: LT52 is glutamatergic and anatomically positioned to inhibit LC10 and the downstream steering cells it drives, motivating the conclusion that LT52 suppresses the reflex to steer toward the fly’s own leg during grooming. The connectome thus converted an otherwise unannotated cell into a node with defined pre- and post-synaptic partners, each of which becomes a candidate for the perturbation experiments that would causally test this feedforward-inhibition model. Its contribution here was generative rather than confirmatory, furnishing not just a novel target but the novel hypotheses that guided experimental direction.

Newly defined central-brain cell types have extended these gains beyond the visual system, clarifying how optic lobe neurons and the central brain interact. TuTu neurons had previously been described as interneurons linking the two optic tubercles across brain hemispheres, receiving input from visual neurons tuned to small objects (Hardcastle et al., 2021). Connectomic reconstruction went further, resolving TuTuA neurons into two subtypes with distinct glutamatergic (inhibitory) outputs and, critically, exposing a reciprocal wiring logic that functional recording alone had not revealed: TuTuA1 targets LC10c while receiving input from LC10a, whereas TuTuA2 targets LC10a while receiving input from LC10c (Schretter et al., 2025). This mirror-image connectivity motif immediately suggested a hypothesis that the two subtypes act as a toggle switch, mutually gating information about fly-sized objects carried by LC10 neurons (Ribeiro et al., 2018). The connectome further situated this motif at an unanticipated intersection with alPg aggression-state neurons, yielding the concrete prediction that internal state should modulate this visual toggle. That prediction was subsequently borne out: optogenetically driven behavioral aggression was found to depend on the LC10a/c toggle (Schretter et al., 2025). Here the wiring diagram of the complete connectome did not merely annotate new neurons but also proposed a specific circuit mechanism linking object vision to behavioral state that was then experimentally verified.

In other instances, the connectome plays a more contextual role. Rather than serving as a source of new hypotheses, it acts as a reference layer that validates experimentally defined cell types and fills in their circuit context, confirming upstream and downstream partners and occasionally pointing to related functional areas. Antibody staining and a genetically encoded dopamine sensor identified two previously undescribed dopaminergic neurons, SIFa and DNp32, whose antennal-lobe innervation the connectome then confirmed. A parallel connectomic search recovered two locomotion-associated PPM neurons that also innervate the antennal lobe. Because DNp32 responds to starvation and more strongly in mated females, these findings hint that state-dependent behaviors may reach odor processing through dopaminergic modulation (Avishai and Parnas, 2025). Likewise, DopaMeander neurons, forward-locomotion-initiating cells identified in optogenetic screens, were matched to their connectome counterparts to confirm that these specific cells, and not others in the driver line, activate walking (Dahlhoff et al., 2025). IR8a neurons, defined by receptor expression at the sensory periphery and shown to promote moisture avoidance in water-satiated flies, were cross-referenced against the connectome to verify connectivity with known hygrosensory glomeruli and expose upstream convergence of parallel hygrosensory pathways (Chu et al., 2024). In each case the connectome functions as an added layer of verification, anchoring experimentally defined neurons within known circuitry and, in addition, hinting at new mechanisms yet to be tested.

## 3 Circuits (at synapse-level resolution)

### 3.1 Sensory circuits

Neuroscience increasingly views the brain as a hierarchy of neural circuits. These networks are composed of interconnected neurons and synapses that carry out well-defined computations. Circuits can be anatomically discrete, like a microcircuit in the hippocampus, or functionally distributed across regions, dynamically recruited as behavioral demands change. Rather than serving as rigid modules for single tasks, they typically contribute in overlapping ways to behaviors such as locomotion, perception, and communication. Prior experimental tools such as electrophysiology, genetic labeling, and calcium imaging offered only partial access to these structures, typically sampling a small fraction of cells or lacking synaptic resolution. With the emergence of microscale connectomes, which map every neuron and synapse, it has become possible to delineate these circuits with synaptic precision and to analyze the network motifs (feedforward, feedback, and recurrent loops) that underlie their function.

This fine-grained view allows researchers to trace how raw sensory signals from the periphery are transformed into increasingly abstract representations of the external world. Before connectomics, one of the clearest mechanistic insights of this type came from studies of motion detection in the *D. melanogaster* optic lobe, where computations converting light input into motion signals were worked out using physiology and genetics (Borst et al., 2010; Borst and Groschner, 2023). Even this well-developed understanding was reshaped by an early, partial connectome. The pioneering dense reconstruction of the medulla motion circuit predated any whole-brain dataset, yet it did more than confirm known wiring: by resolving the synaptic inputs onto T4 and T5 at scale, it exposed the specific circuit architecture that constrained and sharpened competing models of direction selectivity, generating concrete, synapse-level hypotheses that subsequent physiology set out to test (Takemura et al., 2013). Much of the mechanistic work that followed can be read as the experimental unpacking of predictions this reconstruction made possible.

That trajectory is visible across later studies, though the connectome plays different roles in each. In some cases it serves a confirmatory function. Mapping the synaptic distribution of Tm1, Tm2, Tm4, and Tm9 inputs onto T5 dendrites in the FAFB connectome provided the structural foundation for a mechanism that physiology then established, showing that input-specific nicotinic and muscarinic acetylcholine receptors contribute to OFF motion selectivity at single-synapse resolution (Samara et al., 2025). Here the wiring diagram anchored and validated a receptor-level model rather than proposing it.

In other cases the reconstruction was itself the source of the hypothesis. Connectomic analysis of the lobula plate revealed an unexpected architecture that reframed how motion opponency is thought to be computed: rather than being implemented at a single circuit level, opponent inhibition is distributed across three hierarchical levels, comprising T4/T5 terminals, LPi interneurons, and lobula plate tangential cells, with each level predicted to operate at a different spatiotemporal scale to filter directional noise while preserving sensitivity (Ammer et al., 2023). This distributed scheme was not anticipated by prior functional models, but rather the anatomy proposed it. Similarly, reconstruction of hundreds of T4 and T5 neurons across the FAFB medulla generated a structural predictor of a functional property, revealing that the morphological asymmetry of their dendrites predicts preferred direction, and further proposed that the global map of directional selectivity across the visual field is shaped by the geometry of the compound eye (Zhao et al., 2025). Both claims are hypotheses that emerged directly from the wiring and its geometry, offered up for functional testing.

Beyond motion vision, connectomes have expanded the possibilities for fine-grained understanding of other visual tasks, though the role the connectome plays ranges from generating novel hypotheses to confirming ones derived from other approaches. At the generative end, systematic reconstruction of photoreceptor circuitry in the FAFB volume revealed previously unrecognized cell types and connections, including synapses outside the medulla and direct R7-to-L3 contacts, that suggested a candidate R7→L3→Mi9→T4 pathway later tested with calcium imaging and cell-type silencing to establish how color enhances motion detection (Kind et al., 2021; Longden et al., 2023). A connectome-constrained model can also generate unexpected predictions. Investigating hue selectivity, the mechanism by which certain neurons respond to color rather than brightness, a circuit model built from the FAFB wiring predicted, counterintuitively, that recurrent connections are critical for generating hue-selective responses, a prediction then confirmed by targeted genetic perturbation of this recurrence in adult flies (Christenson et al., 2024). In a more confirmatory role, the connectome can be queried to identify the anatomical substrate of a computation hypothesized on other grounds. Analysis of natural scenes together with physiology predicted that stable contrast processing requires a wide-field neuron that pools luminance and inhibits Tm9 with subsequent interrogation of the FAFB connectome identifying Dm12, a glutamatergic cell spanning roughly three medulla columns (∼15° of visual space) and receiving major L3 input, as a candidate matching this profile that was then validated optogenetically (Gür et al., 2024).

Beyond vision, connectomes have been applied across sensory modalities, though here they have more often served to complete and confirm circuit models than to generate wholly unexpected ones. In several cases the wiring diagram primarily fills in an anatomy whose broad organization was already anticipated. Taste circuits have been traced from peripheral gustatory receptor neurons (GRNs) through local interneurons to projection neurons in the sub-esophageal zone (SEZ), and combining the whole-brain connectome with functional calcium imaging showed that taste-quality representation remains largely segregated by modality at these early stages, with most SEZ local interneurons and superior-protocerebrum-projecting neurons predicted to encode a single taste modality (Li et al., 2025; Walker et al., 2025). Circuits for mechanosensation reconstructed from the FAFB connectome similarly confirmed and refined an expected somatotopic organization of head grooming circuits (Eichler et al., 2024; Calle-Schuler et al., 2026). In the olfactory, hygrosensory, and thermosensory systems that converge on the antennal lobe (AL), systematic reconstruction has likewise resolved circuit detail beyond the reach of light microscopy, revealing more projection neuron types and connectivity motifs, including axo-axonic interactions and cross-modal local-neuron computation, than earlier studies had identified, and showing that these channels share glomerular architecture in the AL but diverge in their projections to the mushroom body (MB) and lateral horn (LH) (Berck et al., 2016; Bates et al., 2020; Marin et al., 2020; Schlegel et al., 2021). These reconstructions bridge sensory input to the learning (MB) and valence (LH) centers of the brain, supplying synaptic-level substrates for learning, navigation, and multisensory integration.

In other cases, the connectome has done more than confirm, using the wiring to discriminate between candidate circuit architectures or to predict functional organization in advance of physiology. Mapping the auditory circuits for courtship song, connectomic analysis distinguished between two competing models of song-feature coding and supported an interconnected, non-hierarchical network of song-mode-selective neurons over segregated parallel pathways (Baker et al., 2022), while reconstruction in the male ventral nerve cord connectome (MANC) resolved the song-production circuit into two nested, largely feedforward pathways that separately generate pulse and sine song (Lillvis et al., 2024). The clearest predictive use comes from the interface between vision and navigation: dense reconstruction of the anterior visual pathway in FAFB traced how retinotopic visual input in the medulla is routed, through MeTu and TuBu relay neurons, to the ring neurons that supply the ellipsoid body compass, resolving at least ten parallel channels that each convey a distinct region of the visual field into the navigation system (Garner et al., 2024). Because these channels were found to encode coarse-grained visual features and to disproportionately sample the upper visual field, the connectome predicts which aspects of the visual scene the compass privileges, pointing to celestial cues such as skylight polarization as a navigational priority, a functional hypothesis about visual-to-navigational transformation derived from anatomy alone (Garner et al., 2024).

### 3.2 Motor, navigation, and sensorimotor circuits

Connectomic data has been used to dissect the circuit basis of motor functions and motor-driven behaviors, with the connectome’s role ranges from generating mechanistic hypotheses to confirming mechanisms established by other means. Walk-halting illustrates the generative end. Although several neurons that drive halting in *D*. *melanogaster* had been identified, the circuit mechanisms by which they overrule an ongoing walking state remained unclear. Combining connectome-constrained network models with functional experiments revealed two distinct mechanisms: a “walk-OFF” mechanism, in which GABAergic neurons (FG and BB) inhibit specific descending walking commands in the brain, and a “brake” mechanism, in which newly identified excitatory cholinergic BRK neurons in the nerve cord actively arrest stepping. Here the connectome did hypothesis-generating work: *in silico* coactivation models predicted how these halt neurons suppress walking-promotion descending neurons such as P9 and BPN, and mining the connectome for upstream inputs predicted the sensory contexts that recruit each pathway–predictions subsequently confirmed *in vivo*, including experimental validation of the connectome-predicted neurotransmitter identities (Sapkal et al., 2024).

In the steering domain, so far, the connectome has more often explained or confirmed mechanisms first established by other methods. Descending neurons DNa01 and DNa02 had already been implicated in steering by earlier work, and it was *in vivo* whole-cell electrophysiology and optogenetics, not the connectome, that established DNa02 as a driver of large, high-gain turns; the connectome then explained this physiology, showing that DNa02 (unlike DNa01) makes more direct motor-neuron connections and receives direct input from PFL3 goal-direction cells and from visual projection neurons (Rayshubskiy et al., 2025). In a similar vein, the clearest confirmatory case used leg kinematics, calcium imaging, and optogenetics to show that distinct descending neurons drive distinct steering gestures (DNa02 shortening strides on the inside of a turn, DNg13 lengthening them on the outside), then drew on connectome connectivity and predicted neurotransmitter identities to confirm that these experimentally inferred functions were consistent with the underlying wiring, including the finding that the two neurons draw on largely non-overlapping inputs (Yang et al., 2024).

Steering as a motor action has also been placed in the broader context of complex behaviors such as navigation. Steering circuits receive convergent input from the central complex via the lateral accessory lobe, with connectomic tracing revealing direct pathways from central-complex output neurons onto steering descending neurons (Rayshubskiy et al., 2025). Connectomics have been critical in demonstrating how central complex navigation signals are transformed into steering commands (Mussells Pires et al., 2024; Westeinde et al., 2024). Connectomic data identified PFL2 and PFL3 neurons bridging the head direction system to the locomotor system. Armed with connectivity data and neuronal recordings, models could be fit to explain the transformation of an allocentric, world-oriented goal into egocentric, body-oriented motor commands (Mussells Pires et al., 2024; Westeinde et al., 2024). These mechanistic, connectome-derived models draw on analogies from control systems theory, demonstrating the depth of understanding unlocked by connectome datasets (Westeinde et al., 2024).

The interface between sensory and motor systems has also been explored through the lens of connectomics. Previously described studies on locomotor steering showed intriguing connections onto steering descending neurons from visual projection neurons of the optic lobe as well as from mushroom body output pathways associated with reinforcement learning (Rayshubskiy et al., 2025). More direct studies on sensorimotor integration include work on the presynaptic inhibition of leg proprioceptors during self-generated movement (Dallmann et al., 2025). Combining calcium imaging in behaving flies with the ventral nerve cord connectome, this study showed that the axons of movement-encoding “hook” proprioceptors are suppressed during active behaviors such as walking and grooming, but not during passive leg movement, and identified the responsible circuit: a class of GABAergic 9A interneurons that presynaptically inhibit hook axons (Dallmann et al., 2025). Connectomic tracing further showed that excitatory descending neurons driving walking and grooming recruit these 9A neurons in a leg-specific manner, providing a circuit mechanism for context- and leg-specific gating of movement proprioception (Dallmann et al., 2025).

A particularly clear example of the connectome generating, rather than confirming, a hypothesis comes from the analysis of axo-axonic synapses onto descending neurons (Ceballos et al., 2026). Prior work indicated that these synapses are positioned to veto, amplify, or synchronize descending commands. Charting every axo-axonic input onto the 1,314 descending neurons of the male nerve cord (MANC) connectome revealed that this modulatory network lacks a densely interconnected “rich club” of hubs, instead distributing control across many intermediate “broker” neurons, which suggests that motor modules can be gated independently. Critically, mining this wiring diagram generated a specific, testable prediction absent from prior functional work: that an octet of ascending neurons (AN08B098) providing axo-axonic input onto the giant fiber descending neurons (DNp01) should modulate the escape circuit. This connectome-derived prediction was subsequently confirmed experimentally, with immunostaining establishing the neurons’ cholinergic identity and optogenetic activation showing that they increase giant fiber excitability (Ceballos et al., 2026). Although many connectome-guided studies have focused on locomotion and sensorimotor control, the same approach is increasingly revealing neural mechanisms underlying fundamental physiological processes. Using the male ventral nerve cord and whole-CNS connectomes, researchers identified previously uncharacterized spiracle motor neurons and their upstream control circuitry governing respiration during flight (Luo et al., 2026). Mining the connectome identified candidate respiratory neurons, their upstream interneurons, and a previously unknown circuit architecture linking descending flight commands and interoceptive signals to respiratory control. Connectomic analysis further predicted a feedforward pathway linking descending flight neurons to spiracle opening, together with a parallel interoceptive pathway regulating spiracle closure during dehydration. Subsequent optogenetic, electrophysiological, and behavioral experiments verified these predictions, demonstrating how connectomes can reveal circuit mechanisms that coordinate metabolic demand, water balance, respiration, and motor behavior (Luo et al., 2026).

Taken together, these studies have leveraged connectome datasets to further our understanding of the circuit basis of complex sensory, behavioral, and physiological processes. These studies demonstrate the usefulness of connectome datasets to generate new hypotheses on circuit function or complement experimental data to dissect the mechanisms of circuit function. Across these domains, however, a consistent caveat applies: the connectome has been necessary but rarely sufficient. Structural connectivity constrains the space of plausible mechanisms, but it does not by itself determine how a circuit computes. Several inferential gaps occur. The connectome as wiring diagram reports synapse counts, not physiological weights, so anatomical connection strength is only an approximation of functional influence, one that ignores receptor complement, release probability, or postsynaptic integration. Connection sign is not read out directly from structure either. Although neurotransmitter identity can now be predicted from ultrastructure, these predictions are imperfect and still require confirmation before excitation can be distinguished from inhibition. Chemical-synapse reconstructions omit electrical coupling, leaving gap-junction contributions under-represented. And even a complete, correctly signed graph remains static: the same anatomy can support different computations depending on neuromodulatory state, ongoing network dynamics, synaptic plasticity, and behavioral context, none of which are legible from structure alone. These considerations do not diminish the contributions surveyed here but rather provide exciting challenges to improve the functional realism of connectomes. In their current state, connectomes are most powerful not as a stand-alone oracle of function, but as one element of a closed loop with physiology and behavior, in which wiring yields precise, falsifiable predictions that experiment then adjudicates.

## 4 Connectome graphs: analysis, perturbation, and simulation

### 4.1 Analysis

Connectome graphs are among the largest and most intricate networks in biology, and their size alone places them beyond neuron-by-neuron reasoning; network science supplies the abstractions that render them analyzable, comparable, and simulatable at all (Bullmore and Sporns, 2009; Bassett and Sporns, 2017). At the microscale, connectomes provide an obvious set of nodes and edges corresponding to neurons and synapses, and incorporating axon-dendrite directionality and synapse count between connected neurons allows directionality and weight to be applied to the resulting graph. This abstraction is powerful but lossy. Synapse count is a structural proxy for connection strength rather than a direct measure of functional weight; the relationship between the two is poorly characterized and need not be linear, and count alone carries no sign, such that a numerically strong connection may in fact be strongly inhibitory. Sign can be assigned from measured or predicted neurotransmitter identity, distinguishing excitatory from inhibitory connections, but this remains an approximation: predicted identities carry classification error, and a single transmitter may act with either sign depending on the postsynaptic receptor, as with glutamate in *Drosophila*. Even a correctly signed and weighted graph omits the dynamics, neuromodulatory state, and plasticity that ultimately shape function–limitations that bound every analysis and simulation that follows, and that motivate the progression from describing the static graph to activating and, finally, perturbing it.

Applied to the fixed wiring diagram, network science first offers a shared quantitative vocabulary for describing individual connectomes and, in turn, comparing them. The first such descriptions characterized whole-network topology for the *C. elegans* and *D. melanogaster* connectomes (Varshney et al., 2011; Lin et al., 2024). Beyond differences in network size, the *D. melanogaster* connectome shows sparser connection probability but higher average synapse strength than *C. elegans* (Lin et al., 2024). Both display small-world topology meaning low path lengths with high clustering, a configuration associated with efficient inter-node communication (Watts and Strogatz, 1998; Humphries and Gurney, 2008; Lin et al., 2024) with small-world coefficients (*D. melanogaster* SΔ = 141; *C. elegans* SΔ = 3.21) comparable to established small-world networks such as the internet (SΔ = 98.1). Both also exhibit a rich club, in which well-connected neurons preferentially synapse onto one another (Towlson et al., 2013; Lin et al., 2024), though it comprises a far larger share of neurons in *D. melanogaster* (30%) than *C. elegans* (4%). Within the *D. melanogaster* rich club, neurons are classified as broadcasters or integrators by their in- versus out-degree ratio; the thresholds are somewhat arbitrary, yet only *D. melanogaster* contains neurons with such divergent in- and out-degrees. Finally, connection reciprocity and connectivity motifs exceed generative-model expectations in both larval (Winding et al., 2023) and adult *D. melanogaster* (Lin et al., 2024).

These topological summaries are informative but sensitive to how the graph is built. Small-world coefficients, rich-club membership, and motif counts all shift with the synapse-count threshold used to define an edge and with whether nodes represent individual neurons or aggregated cell types; most such metrics are also sign-blind, treating excitatory and inhibitory edges alike. Such descriptive work nonetheless hints at structures underlying nervous system function. In *D. melanogaster*, neuropils with specialized roles deviate from whole-network properties: the mushroom body, a well-established learning center, is unusually rich in integrating neurons and network motifs (Winding et al., 2023; Lin et al., 2024). Relating this structure to function, however, will require perturbation studies (Kulkarni and Bassett, 2025) especially because a structural snapshot cannot capture the very plasticity that reshapes a learning center, whose synaptic weights encode experience rather than fixed anatomy. And while network properties have been described for connectome subvolumes of other species (Lin et al., 2024), whether they scale to whole nervous systems remains unknown.

The payoff of description is comparison. Just as sequence alignment lets genomicists place any two genomes in a common frame and read off precisely where they differ, comparative connectomics seeks to distinguish universal principles of neural wiring from species-specific adaptations (Van Den Heuvel et al., 2016). Realizing this at scale will require principled methods to align connectomes and quantify structural divergence–between individuals, across species, and between healthy and diseased brains. Here connectomics can inherit a maturing toolkit: in network biology, network alignment already plays the role for molecular interaction networks that sequence alignment plays for genomes, transferring functional annotation between well- and less-studied species across aligned subnetworks (Lázaro et al., 2025). Much as genetics moved from candidate-gene hypotheses to genome-wide quantification across thousands of SNP and QTL sites, connectomics may move from candidate-circuit hypotheses to connectome-wide quantification of network perturbation sites.

Beyond description and comparison, network science also functions as an experimental instrument. By reducing network dimensionality, community-detection methods make otherwise intractable connectomes designable for experiments. Hierarchical Louvain clustering of descending neurons (DNs) in the *D. melanogaster* connectome, for instance, yielded clusters corresponding to discrete behavioral patterns, complementing optogenetic and imaging experiments to propose models of DN recruitment during action selection (Braun et al., 2024). Connectivity clustering has likewise been used to match neurons across datasets the sex-specific VNC connectomes and emerging full-brain connectomes (Stürner et al., 2025). In each case the clustering generates hypotheses that physiological experiments then test, rather than standing as functional claims on their own. Community detection can likewise be applied to a deliberately restricted subgraph to expose organization that whole-brain clustering obscures: modularity maximization over the input neurons of the oviposition inhibitory neuron–itself among the most highly connected integrator neurons in the Hemibrain–resolved seven candidate input pathways that did not match the neuropil-scale modules recovered from the full connectome, and that the authors treat as hypotheses for circuit discovery rather than established circuits (Weber-Langstaff et al., 2025). These methods characterize, compare, and organize the connectome, but they operate on a static substrate; to ask what a circuit does rather than how it is wired, dynamics must be placed on the graph.

### 4.2 Simulation

Much as other fields have benefited from digital twins, neuroscience would gain immensely from an *in silico* brain in which experiments could be run rapidly and with reliable results. The wiring diagram supplies a necessary foundation for such models, though not a sufficient one: a connectome-based simulation is a twin of the wiring, run under assumed dynamics in a single state, rather than of the living, neuromodulated, plastic brain. The connectome is in this sense widely regarded as necessary but not sufficient for understanding function–a framing spanning views that range from the connectome as only a weak constraint to the connectome as the proper starting point for brain simulation (Bargmann and Marder, 2013).

Even simple leaky integrate-and-fire (LIF) models have gained new predictive power once the connectome supplies brain-wide simulation parameters (Shiu et al., 2024). Running such models, however, requires importing precisely what the connectome omits: connection sign, here inferred from predicted neurotransmitter identity, whose classification errors propagate into the simulated dynamics; and synaptic weight, scaled from synapse count under an assumed and likely non-linear mapping. Point-neuron LIF dynamics further discard dendritic integration and intrinsic excitability, bounding such models to predictions at the population rather than single-cell level.

A complementary approach builds neural networks constrained directly by connectomic wiring (Lappalainen et al., 2024). Mimicking the connectivity of the *D. melanogaster* optic lobe, such a network–with architecture fixed by the connectome while single-neuron and synaptic parameters were optimized against a functional objective–predicted novel motion-sensitive neurons from moving-image input (Lappalainen et al., 2024). That anatomy alone did not suffice, and that tuned parameters were required to recover function, is itself among the clearest demonstrations that connectome structure constrains dynamics without determining them.

Experimental validation of simulation predictions is ongoing, and a growing number of studies pair simulation with experiment directly–in some cases using the connectome not to recapitulate known circuits but to generate genuinely novel hypotheses. Firing-rate models implemented in the male VNC connectome enabled computational screens of neurons downstream of DNg100, a descending neuron whose optogenetic activation is sufficient to trigger walking (Pugliese et al., 2025). Beyond identifying a three-neuron central pattern generator for rhythmic leg movement, the screen predicted rhythm-generating neurons downstream of DNb08–a previously untested descending neuron–whose optogenetic activation then confirmed rhythmic leg movements distinct from those of walking (Pugliese et al., 2025). Here the model did not merely reproduce an established result; it nominated a specific, untested neuron for a specific function that experiment subsequently bore out. Such confirmations mark the essential step: a connectome-based simulation is a hypothesis until validated against physiology, and evidence thereafter.

Further integration extends these neural simulations into the body they must ultimately control. Because behavior emerges from the interplay of neural dynamics, biomechanics, and the physical environment, connectome-derived circuits can be coupled to morphologically realistic body models–such as the MuJoCo-based whole-body model (Vaxenburg et al., 2025) and the NeuroMechFly platform (Lobato-Rios et al., 2022; Wang-Chen et al., 2024)–that place a simulated nervous system inside a simulated body within a physics engine. Embodiment offers a route to more complete validation: a predicted locomotor circuit such as the DNb08 pattern generator could be judged not only by whether it fires rhythmically, but by whether it drives realistic movement in a closed sensorimotor loop. NeuroMechFly’s latest iteration already couples such a body to a connectome-constrained visual network (Wang-Chen et al., 2024), indicating that the neural and biomechanical strands of connectome-based modeling are beginning to converge.

### 4.3 Perturbation

New techniques for graph perturbation, whether *in silico* by editing the connectome graph directly or *in vivo* by targeting the neurons and synapses it represents, promise for neuroscience what genome editing gave genetics: the ability to move beyond observation and deliberately intervene, testing hypotheses about brain function by altering connectivity and observing the result (Kulkarni and Bassett, 2025). Whereas sequencing revealed which genetic elements were present, editing let genetics ask what each element does by changing it; connectome perturbation offers the analogous leap from mapping connectivity to interrogating it causally–though at present the *in vivo* manipulation of individual synapses at scale remains aspirational relative to the *in silico* case.

This progression is already underway. *In silico*, a whole-brain leaky integrate-and-fire model supports systematic perturbation of the connectome graph, silencing neurons to test their necessity and activating them to test their sufficiency, with predictions validated against genetic silencing and optogenetic activation in living flies (Shiu et al., 2024). Looking toward *in vivo* intervention, the goal has been formalized as moving from the connectome–which specifies which neurons can affect one another–to the “effectome,” a causal model of how strongly they actually do, recoverable only by perturbation (Pospisil et al., 2024). Casting optogenetic stimulation as an instrumental variable and using the sparse connectome as a Bayesian prior, this framework renders whole-brain causal estimation tractable, and an eigendecomposition of the connectome nominates the compact circuits whose perturbation would most constrain brain-wide dynamics, recovering known circuits such as an opponent-motion detector and a ring-attractor winner-take-all. Together these mark the two frontiers of connectome perturbation: *in silico* editing that is already routine, and a principled path toward the targeted *in vivo* perturbations that would let the connectome be read not merely as a map of what connects to what, but of what changes what.

Across analysis, simulation, and perturbation, network science serves less as a single method than as the layer through which connectomes become tractable objects of study–described, compared, simulated, and perturbed. As more connectomes are published, these tools will be what integrates them into a comparative and generative science, addressing questions of neurodevelopment and circuit function across individuals, species, and disease states (Barabási et al., 2023).

## 5 Annotation and cross-reference

Studies reviewed so far have used connectome datasets to guide experimental design or computational analyses. Yet many studies have not used connectome datasets directly but have checked experimental results against the connectome as a reference, pointing readers to entries in the connectome database for further reference (Chu et al., 2024; Cowley et al., 2024; Avishai and Parnas, 2025; Bervoets et al., 2025; Valentino et al., 2025). Other studies have used their experimental results to annotate neurons in the metadata section of connectome datasets (Lapraz et al., 2023). These studies show that even when not directly used for experimental design, connectomes can provide both a sanity check for experimental results and a platform for collaborative data sharing. EM reconstruction has retrospectively corroborated structure–function hypotheses derived from physiology and modelling (Turner-Evans et al., 2020) and cell types first defined by Golgi anatomy (Fischbach and Dittrich, 1989; Nern et al., 2025) while comparison between independent connectomes has validated the stereotypy of most cell types and shown that roughly a third of hemibrain types could not be reliably re-identified in FlyWire (Schlegel et al., 2024). Such checks are not automatically confirmatory (Braun et al., 2024), but they are only possible because the underlying data and infrastructure are openly shared (Zheng et al., 2018; Dorkenwald et al., 2022; Plaza et al., 2022; Clements et al., 2024).

Comprehensive experimental resources in *D. melanogaster* such as the FlyLight split-GAL4 lines provide an excellent platform to cross reference with connectome datasets (Meissner et al., 2025; Shuai et al., 2025). Many of these lines target a small population of neurons in *D. melanogaster* (sometimes only one neuron), and in combination with a UAS line may be embedded with an optogenetic sensor through which experimenters may force activation or silencing of these neuron populations. Optogenetic screens can then refine new target neurons for researchers to analyze (Henning et al., 2026). Matching these neurons to connectomic datasets by morphological search (Clements et al., 2024), now built into the major driver line collections (Meissner et al., 2025; Nern et al., 2025), yields their synaptic connectivity (Plaza et al., 2022) and predicted neurotransmitter identity (Eckstein et al., 2024).

## 6 Comparative connectomics

Fly connectomics is a rapidly advancing field, having recently and rapidly moved beyond a single individual (*n* = 1) for *Drosophila melanogaster* (Stürner et al., 2025), and, similar to earlier work on *C. elegans* connectomes, fly connectomics now encompasses comparisons and differences across the sexes. For example, the recent publication of the full male connectome enabled comparisons with previous female connectomes (Berg et al., 2025). Importantly, such comparisons move connectomics beyond anatomical description and toward understanding how variation in neural wiring contributes to variation in behavior, providing a framework that can eventually be extended to age, physiological state, disease, and species-level comparisons. Raw inputs among male and female brains were found to be stereotyped, while higher order brain regions demonstrated differential connectivity (Berg et al., 2025). Beyond additional possible future connectomes completed for *D. melanogaster*, such as increases to sample size for confirmations, or across age, physiological state, learning and memory treatments, as well as disease modelling, it is likely that connectomes in the future will become available for more species within this genus. Importantly, *Drosophila* as a model genus provides an ideal neural universe to explore with connectomics, especially given that within this one genus you have highly diverse host preferences (e.g., fresh vs rotten fruit, flowers, mushrooms, cacti, tree sap, land crabs, bat guano, generalists vs specialists) as well as diverse habitats or geography (e.g., mountain, desert, rain forest, island, mainland) with these fly species found on every continent except Antarctica. This genus also already has strong genomic resources across nearly 100 species, as well as firmly established phylogenetic and evolutionary timescales, although physiology, behavior, and functional neuronal data, are notably lacking for most species within the genus. Moreover, for many species within the *Drosophila* genus, genomic resources now substantially outpace detailed neurobiological and ecological or natural history characterization.

Moreover, as a wider range of other arthropod connectomes are published, it will be possible to compare datasets to quantify stereotypy among species connectomes and compare evolutionary change in brain connectivity across larger phylogenetic boundaries such as insect orders, like across Diptera (true flies), Coleoptera (beetles), Lepidoptera (butterflies and moths), Orthoptera (crickets and grasshoppers) and Hymenoptera (ants, bees and wasps). Comparative connectomics is also beginning to expand beyond insects. In vertebrates, synapse-resolution reconstructions have now been produced for larval zebrafish, including a whole-brain dataset comprising approximately 0.058 mm^3^ of neural tissue, providing the first vertebrate connectome at this scale and resolution (Svara et al., 2022). More targeted reconstructions have also emerged in birds and mammals. In the zebra finch, dense EM reconstructions from Area X of the song system have enabled analyses of neuronal migration and adult neurogenesis within a connectomic framework (Shvedov et al., 2026). In mammals, connectomics has thus far focused primarily on local microcircuits and cortical subvolumes, exemplified by reconstructions from mouse visual cortex and related datasets. Beyond vertebrates, partial connectomes have also been generated in evolutionarily distant systems, including the learning and memory circuitry of the octopus vertical lobe, providing an important comparison for understanding the convergent evolution of complex cognition and neural architectures (Bidel et al., 2023). Together, these studies demonstrate that connectomics is increasingly moving beyond a handful of traditional model systems, providing the foundation for future comparative analyses spanning deep evolutionary timescales. However, some insect orders will be substantially more challenging than others. For example, the European honeybee (*Apis mellifera*; Hymenoptera) is estimated to possess over 1 million neurons, representing roughly an order-of-magnitude increase in neurons over current *D. melanogaster* complete connectome efforts. For an evolutionary perspective, recent work has reconstructed larval *D. melanogaster* and *D. erecta* antennal lobes (Roberts et al., 2025). Here the ecology of both species varies from the ultimate fruit generalist in *D. melanogaster* and the pandanus fruit specialist in *D. erecta*. Consequently, the antennal lobe comparison revealed broad stereotypy in both species, but with distinct bottlenecks or hotspots of evolutionary rewiring (Roberts et al., 2025). These types of studies in the near future will be a critical test of connectomics, especially related to our understanding of the freedoms and constraints regulating brain evolution. By identifying not only where circuits differ, but also where they remain remarkably conserved despite ecological divergence, comparative connectomics across species may reveal which aspects of nervous system architecture are most evolutionarily flexible and which are fundamentally constrained, while also providing a framework or justification for targeted, partial EM connectomes within close relatives to test specific hypotheses.

Importantly, the scientific questions addressed by fly connectomic analyses often differ between the central brain and ventral nerve cord. Brain connectomes have primarily been used to investigate sensory integration, learning, navigation, and higher-order information flow, whereas VNC connectomes have been particularly valuable for understanding locomotion, motor coordination, descending control, and sensorimotor transformations. Together, these complementary datasets provide a systems-level view of how sensory information is processed, transformed, and ultimately converted into behavior.

Future comparative studies will likely find hotspots of evolutionary change or stereotypy breaks, allowing researchers to focus on these areas, such as through targeted studies of specific brain areas or neuron cell types. As summarized in Figure 4, whole-brain connectomes are becoming increasingly available for smaller organisms, and partial connectomes are becoming increasingly available for larger organisms. As depicted in Figure 4, there will be a need for both vertical growth–more complex connectomes–and horizontal growth–more replicates at a given level of complexity. As an emerging discipline, comparative connectomics would likely require an analogous toolkit to the one currently available for comparative genomics (Van Den Heuvel et al., 2016). Again, these are exciting times for comparative neuroethology, as the tools are increasingly in place for this deep and exciting level of detailed comparison!

**FIGURE 4:**
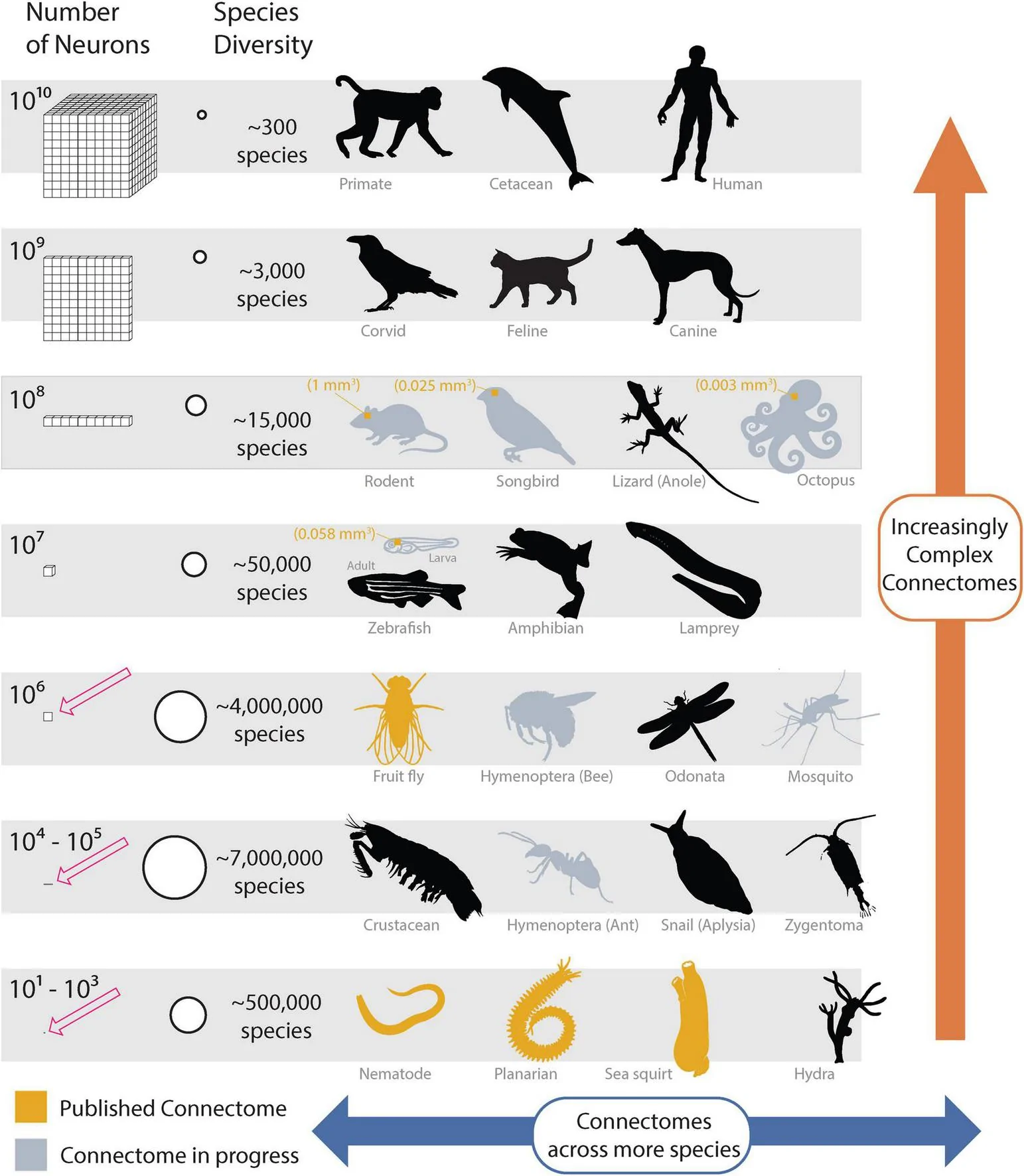
Two complementary directions for the future expansion of connectomics. Connectomic datasets can expand along two largely independent axes. Horizontal expansion (blue arrow) represents increasing taxonomic breadth and replication, including additional individuals, populations, sexes, developmental stages, treatments, disease models, and species at a similar level of nervous system complexity. Such datasets are particularly valuable for comparative connectomics, population variation, and evolutionary analyses. Vertical expansion (orange arrow) represents the extension of connectomic methodologies toward increasingly complex nervous systems containing larger numbers of neurons and synapses, progressing from simple invertebrates to vertebrates and ultimately mammalian and human brains. Representative species are shown according to approximate nervous system size and broader species diversity within each lineage. Colors indicate whether synapse-resolution connectomes have been published (yellow) or remain in progress (grey). Examples of intermediate vertebrate and non-traditional model systems include the larval zebrafish whole-brain connectome (∼0.058 mm^3^ reconstructed, excluding the retinae), the partial zebra finch song-system (∼0.025 mm^3^; Area X), the octopus vertical lobe connectome (∼0.003 mm^3^), and mouse cortical connectomes, including a 1 mm^3^ reconstruction of visual cortex. Although current vertebrate connectomics are largely restricted to local circuits, subregions, or partial nervous systems, continued advances in electron microscopy, light-based imaging, molecular barcoding, and artificial intelligence may ultimately enable simultaneous progress along both the horizontal and vertical axes. Such developments would allow connectomics to move from single exemplar datasets toward a broader comparative, population-scale, and evolutionary science analogous to modern genomics.

## 7 Discussion

External scientific perspectives on the field of connectomics might rightfully point out the low number of replicates or individuals currently available. Although no longer an n of 1, for *Drosophila melanogaster* we only have a few full and complete adult connectomes (see Figure 2). However, one of the most important insights from connectomics has been the unbelievable symmetry of brain hemispheres at the microscale. Thus, several analyses have successfully used the hemibrain as the unit of analysis (Scheffer et al., 2020), meaning that by dividing our full connectomes and combining them with the original hemibrain dataset, the use of independently reconstructed hemispheres effectively increases the number of available comparison units, although these should not be interpreted as fully independent biological replicates (e.g., pseudoreplication). Notwithstanding this new form of analysis, there are still a notably low number of individual replicate connectomes in both larvae and adult *D. melanogaster*. As technological advances proceed and allow the generation of new connectomes at record speeds, we will be more certain about analyses conducted on earlier datasets, and as sample sizes grow, more avenues for comparative neurobiology will continue to emerge.

With the advent of more connectome datasets, another emerging challenge will be the storage, standardization, and indexing of connectomes. In this review we have referenced connectomes using popular acronyms (Figure 2), yet this practice will become obsolete as new connectomes require the assignment of unique and systematic identifiers. Moreover, connectomes generated by different research groups have so far used similar but different schema to classify and organize both neurons and synapses. Moving forward, there will be a pressing need for connectomes to have standardized schema with room for metadata about the underlying dataset such as the species, developmental stage, or sex of the imaged organism.

These challenges mirror the emergence of genomic datasets, where small, early DNA sequences could be written in lab notebooks, whereas full genomes required the formation of resources such as GenBank to store and classify an increasing number of sequences. These challenges will likely be even more substantial for connectomes since the underlying imaging datasets require orders of magnitude greater storage space compared to genomes. Moreover, storage needs will scale up considerably more for vertebrate connectomes such as zebrafish, mouse, or human connectomes. Current vertebrate connectomics largely remains restricted to local circuits, subregions, or partial nervous systems, as illustrated by partial reconstructions in mouse cortex, the zebra finch song system, and the octopus vertical lobe (Svara et al., 2022; Shvedov et al., 2026; Bidel et al., 2023). As an example, a mouse genome could be stored easily in a single modern laptop whereas the entire mouse connectome is estimated to require an exabyte of storage capacity (Abbott et al., 2020), roughly equivalent to 1 million modern laptops (Figure 5). As these technical challenges are addressed and new technologies allows faster, cheaper and more automated connectome reconstruction, there will be a need to target a broader swathe of the tree of life. As Figure 4 shows, there is a tremendous wealth of species at the current nervous system size we can reconstruct (∼100,000 neurons). These developments mirror the advancements in genomics that allowed the sequencing of thousands of genomes for diverse species.

**FIGURE 5:**
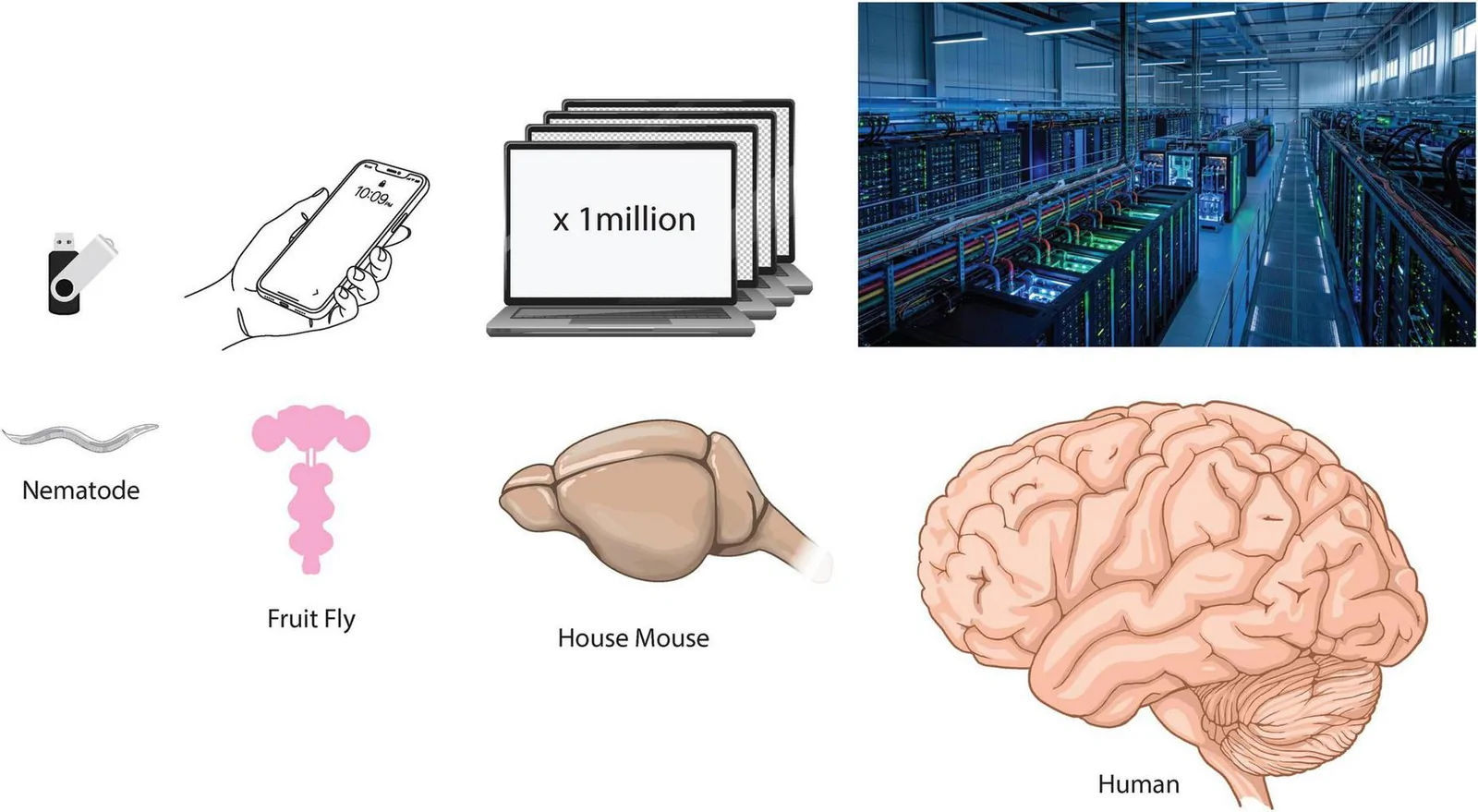
Scaling challenges in data storage associated with connectomes across organisms. While smaller models such as *C. elegans* (with 302 total neurons) can have their connectomes stored on a flash drive, these storage requirements scale upward rapidly and exponentially. For example, the datasets for *D. melanogaster* (< 200,000 neurons) can fit in roughly the storage of a current generation smart phone, while estimates for a mouse (∼70 to 100 million neurons across entire nervous system) would take around 1 million laptops or mobile workstations, and a human storage estimate is closer to a computer cluster or that of an entire data center (current estimates ∼86 billion neurons in human central brain).

Another important critique is the static nature of connectome datasets. Some structural information such as mitochondria distribution may yield insights into function prior to imaging, yet our current techniques require destructive slicing and fixation of sample brains. Thus, we cannot follow the formation and rewiring of an organism’s nervous system across a lifetime. With future replicates of genetically similar individuals, we may yet provide a statistical view on connectome formation and experience dependent rewiring (Trachtenberg et al., 2002; Xu et al., 2009). With regards to functional connectomes, much work remains to further use functional datasets to interpret static connectomes. As indicated in this review, connectome databases can serve as a platform to crosslink and cross reference functional datasets. Connectomes are particularly suited as a hub dataset to further integrated neuroscientific datasets, which will shed light on both the detailed structure and detailed function of nervous systems. Perhaps the best integration of functional and connectome datasets remains the MICrONs mouse visual cortex connectome, where researchers performed *in vivo* calcium imaging on the individual mouse prior to connectome imaging, allowing correlation of both datasets (The MICrONS Consortium et al., 2025). This approach of functional data collection right before connectome imaging may be particularly important for future connectomes, as functional datasets and structural connectome datasets will have a one-to-one mapping. In this way, connectomes move beyond serving as static wiring diagrams and instead become a hub or linking dataset through which diverse neuroscientific measurements can be organized, unified and interpreted within a common cellular framework. More broadly, we envision connectomics progressing through a continuum of graph analysis, graph simulation, and graph perturbation, analogous to the progression from sequence description to genome engineering in genomics.

What remains obvious is that technological advances will be key for the progression of connectomics as a scientific field (Figure 6). It was only through the advent of computer vision technology that the *D. melanogaster* connectome became a feasible target for reconstruction, and now with repeated sampling across several individuals. Even so, extensive human proofreading in the span of decades was still required to generate a first, quality connectome for this invertebrate. As artificial intelligence (AI) reconstructions advance, there may be less need for human proofreading, allowing researchers to confidently use either purely AI reconstructed connectomes or slightly human proofread versions, which drastically cuts time and cost. On the imaging front, the most promising developments for lowering connectome reconstruction costs have been the emerging use combined light based, expansion microscopy and multiplex, neuron barcoding methodologies (Bosch et al., 2025; Tavakoli et al., 2025; Zhong et al., 2025; Chen et al., 2026). Advancements in both imaging and AI aspects of connectome development, methodologies, and reconstructions may one day allow researchers to reconstruct connectomes using standardized kits like the ones currently used by genomics researchers. Just as inexpensive genome sequencing transformed the field of genetics from a gene-by-gene discipline into a comparative and population-scale science, in the future, routine connectome reconstruction may eventually enable large-scale comparisons across individuals, developmental stages, physiological states, species, and disease conditions. Again, this is an exciting time for neuroscience, and the future looks bright based on this recent resurgence of connectomics.

**FIGURE 6:**
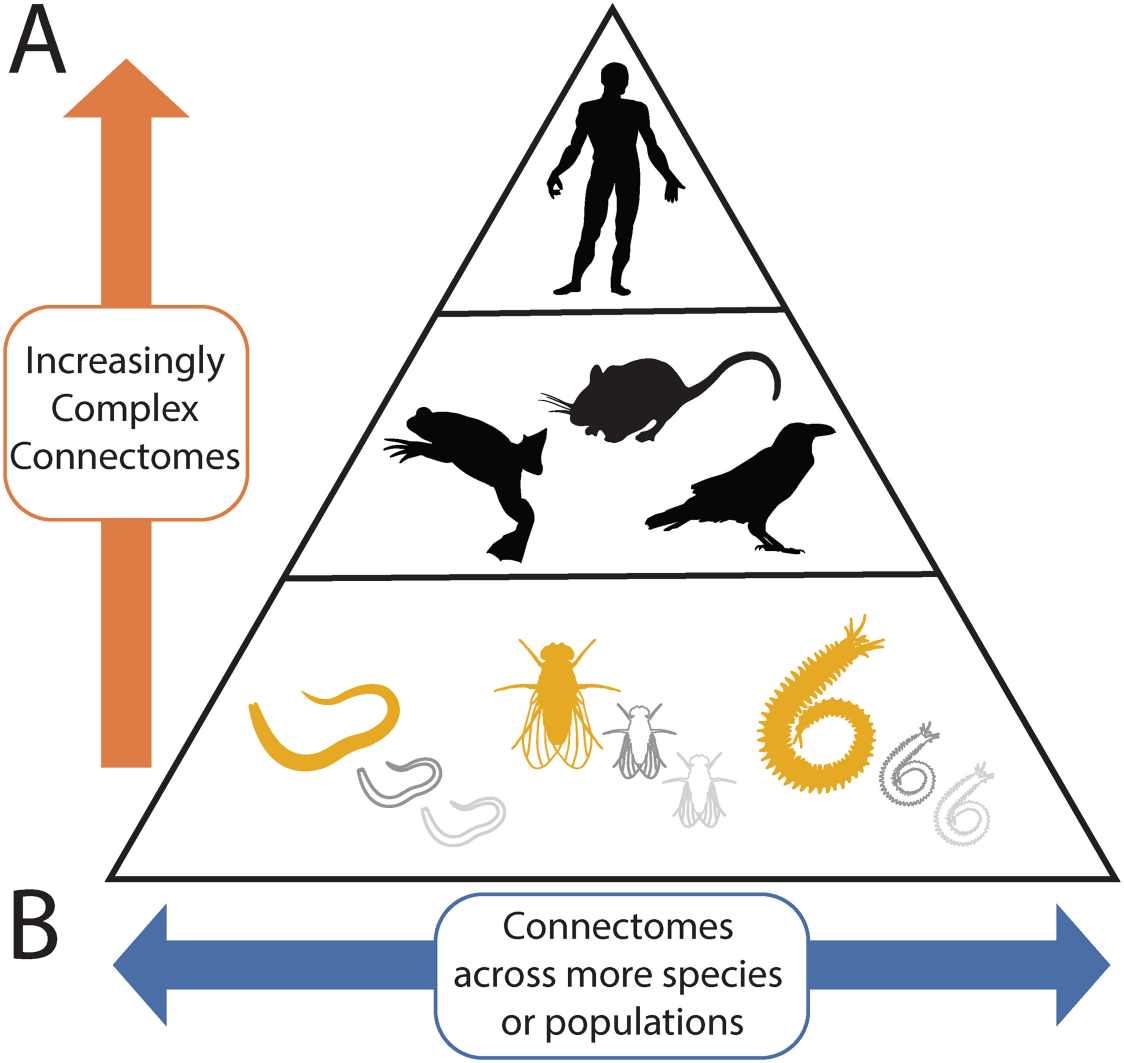
Two complementary directions for the future growth of connectomics. **(A)** Vertical expansion aims to push the limits of feasibility toward increasingly complex nervous systems, from invertebrates to vertebrates and eventually mammalian and human brains. **(B)** Horizontal expansion aims to move connectomics from single exemplar datasets toward a comparative science by reconstructing additional individuals, populations, treatments, developmental stages, and species. Whereas vertical expansion addresses questions of scale and complexity, horizontal expansion enables investigation of variation, evolution, development, learning, disease, and neural diversity across the tree of life. Much as large numbers of genomes transformed genetics into a comparative and popul bbation-scale discipline, increasing numbers of connectomes may ultimately transform neuroscience into a comparative and evolutionary science of nervous system organization. The future of connectomics may therefore depend not only on larger connectomes, but also on substantially more connectomes.
